# Symbiosis of Arbuscular Mycorrhizal Fungi and *Robinia pseudoacacia* L. Improves Root Tensile Strength and Soil Aggregate Stability

**DOI:** 10.1371/journal.pone.0153378

**Published:** 2016-04-11

**Authors:** Haoqiang Zhang, Zhenkun Liu, Hui Chen, Ming Tang

**Affiliations:** College of Forestry, Northwest A&F University, Yangling, Shaanxi, 712100, PR China; IPK, GERMANY

## Abstract

*Robinia pseudoacacia* L. (black locust) is a widely planted tree species on Loess Plateau for revegetation. Due to its symbiosis forming capability with arbuscular mycorrhizal (AM) fungi, we explored the influence of arbuscular mycorrhizal fungi on plant biomass, root morphology, root tensile strength and soil aggregate stability in a pot experiment. We inoculated *R*. *pseudoacacia* with/without AM fungus (*Rhizophagus irregularis* or *Glomus versiforme*), and measured root colonization, plant growth, root morphological characters, root tensile force and tensile strength, and parameters for soil aggregate stability at twelve weeks after inoculation. AM fungi colonized more than 70% plant root, significantly improved plant growth. Meanwhile, AM fungi elevated root morphological parameters, root tensile force, root tensile strength, Glomalin-related soil protein (GRSP) content in soil, and parameters for soil aggregate stability such as water stable aggregate (WSA), mean weight diameter (MWD) and geometric mean diameter (GMD). Root length was highly correlated with WSA, MWD and GMD, while hyphae length was highly correlated with GRSP content. The improved *R*. *pseudoacacia* growth, root tensile strength and soil aggregate stability indicated that AM fungi could accelerate soil fixation and stabilization with *R*. *pseudoacacia*, and its function in revegetation on Loess Plateau deserves more attention.

## Introduction

Loess Plateau, which located in the semi-arid region of center China, has become one of the most severely eroded areas in the world due to frequent heavy summer rain storms, steep landscapes, long-term human activities (since the 15th century), and highly erodible soils [1]. Revegetation had been reported as one of the most effective ways to reduce soil and water erosion on Loess Plateau [1, 2]. In the process of revegetation, *Robinia pseudoacacia* L. (black locust) was widely planted on Loess Plateau for revegetation to control soil erosion since 1950s [3, 4]. *R*. *pseudoacacia* was used as a pioneer tree species due to its fast growth and strong capacity in improving soil nitrogen content and availability, available phosphorus pool, organic carbon sequestration as well as soil chemical and microbiological properties [2, 5–9]. Also, the choice of *R*. *pseudoacacia* in soil erosion control on Loess Plateau was because of its important economic value: it produces durable and rot-resistant wood as well as honey [10]; it can be used in coppice system and as fodder in silvopastoral system [11]; it has the potential for bio-oil production and fuel ethanol derived from biomass [12–14].

Arbuscular mycorrhizal (AM) fungi belong to the phylum Glomeromycota and can form mutualistic symbiosis with more than 80% terrestrial plants [15, 16]. With the established symbiosis, AM fungi acquired carbon from host plant and in return supplied host plant with water and mineral nutrients [17–20]. In order to maintain the nutrient exchange, AM fungi usually produced a large amount of extraradical hyphae to explore soil, and transferred water and mineral nutrients into inter- and intra-radical hyphae which extensively expanded inside plant roots [16]. In this process, AM fungi produced a large amount of hyphae, and changed root biomass as well as root morphology [21–24]. The accumulated AM fungal hyphae inside soil could also produce a protein or protein class quantified as glomalin-related soil protein (GRSP) [25–27]. Combined the effect of AM fungal hyphae and GRSP, the formation of soil aggregate, which is important in resistance of soil erosion, was promoted [28, 29]. Besides soil aggregate, plant root tensile strength is one of the most important factors for soil stabilization [30–32]. In previous studies, the root tensile strength was mostly compared among different plant species [32, 33]. But the influence of AM fungi on root tensile strength should not be neglected, because the fundamental (morphological, physiological, biochemical) change introduced by AM fungi [16].

In the study of *R*. *pseudoacacia*, AM fungi could colonize this legume woody tree [34, 35]. However the influence of AM fungi on *R*. *pseudoacacia* in control of soil erosion on Loess Plateau was rarely studied. With the aim to investigate the effect of AM fungi, we carried out a pot experiment with two AM fungi, and assessed (1) the relationship between AM fungi and *R*. *pseudoacacia* seedling, (2) the effect of AM fungi on the plant growth, root morphological characters, and root tensile strength, (3) the effect of AM fungi on GRSP content and soil aggregate stability, (4) the relationship among plant root, AM fungal hyphae, GRSP content, and soil aggregate stability. We hypothesized that the AM fungi could increase (1) the root tensile strength, and (2) the soil aggregate stability, consequently increase the capability of *R*. *pseudoacacia* in control of soil erosion.

## Materials and Methods

### Plant material and soil preparation

The soil used in this study was collected from the top layer of Northwest Agriculture and Forestry University (http://www.nwsuaf.edu.cn/) campus nursery, in Yangling City, Shaanxi province, China. The soil was ground, passed through a 2 mm sieve, autoclaved at 0.11 MPa and 121°C for 2 h, then placed one week before use. The main nutrient characters were as follows: organic matter 11.85 g/kg, available nitrogen 41.25 mg/kg, available phosphorus 9.63 mg/kg and available potassium 134.62 mg/kg, measured as described by Gao [36].

*R*. *pseudoacacia* seeds were purchased from a local market (Yangling, China). Seeds were sterilized with 10% H_2_O_2_ for 10 min, and washed 3 times with sterilized water. Sterilized *R*. *pseudoacacia* seeds were germinated on wet filter paper in petri dishes. Germinated seeds were transplanted into plastic pots of 15 cm in diameter, 16 cm in depth and filled with 1.5 kg autoclaved soil. Each pot was planted with three seedlings and only one seedling was kept in each pot after one week growth.

### AM fungi inoculation

*Rhizophagus irregularis* (BGC BJ09) and *Glomus versiforme* (BGC GD01C) were provided by Plant Nutrition and Resources Institute of Beijing Academy of Agriculture and Forestry Science (http://www.baafs.net.cn/index.aspx), and propagated in pot culture with *Trifolium repens* using sterile sand prior this experiment. The result mixture consisting of spores (50 spores/g), mycelium, and colonized root fragments was used as AM fungal inoculum.

For AM fungi inoculation, 20 g inoculum was placed in plastic pot 2 cm below geminated *R*. *pseudoacacia* seeds, while the non-mycorrhizal treatment received 20 g autoclaved inoculum with microbial wash (1-μm nylon mesh) from the inoculum.

### Experiment design and plant growth

In order to test the effect of AM fungi on plant growth and soil aggregate characters, three treatments were set up: RI, inoculated with *R*. *irregularis*; GV, inoculated with *G*. *versiforme*; CK, mock-inoculated. Each treatment had 30 repeats and the plastic pots were randomly arranged. In total, 90 seedlings were grown in pot experiment.

The experiment was conducted in a greenhouse with temperature of 20–35°C, 12–14 h day light, and relative air humidity of 55–78%. Each plastic pot was fertilized with 50 mL Hoaglands’ solution of original concentration [37] for each week and watered with deionized water every day.

### Plant growth, root morphology and AM fungal colonization analysis

*R*. *pseudoacacia* seedlings were harvested 12 weeks after transplanting. Six seedlings for each treatment were analyzed. Seedling roots were carefully washed with tap water to remove all soil particles, and dried with paper towels. Fresh weight of shoot and root was recorded.

The images of roots were stored in the computer via digital scanner (STD1600 Epson USA). Total root length, root surface area, root volume, average diameter, tip number and number of forks were determined, and root was classified (diameter 0–0.2 mm, 0.2–0.5 mm, 0.5–1.0 mm and > 1.0 mm) by the scanner supporting WinRhizo (Version 5.0B) root analysis system software (Regent Instrument Inc, Canada).

After the image scan, roots were separated into three portions. One portion was oven-dried at 80°C until constant weight to calculate root dry weight. One portion was stained with trypan blue [38], and the mycorrhizal colonization rate was measured by the gridline intercept method [39].

One portion of root was used to determine root tensile strength (*T*_r_ MPa). Computer-controlled electronic universal testing machine (WDW-100E, Shanghai Jielun Inc., China) was used and the measuring force was in the range of 0–100 KN. Root samples were adjusted to the fixture about 5 cm, fixed by tightening the clamps. The machine stretched slowly at constant speed of 10 mm/min. During roots stretching process, the data collection instrument obtained data automatically. Each treatment was measured for 80 times. The root tensile strength (*T*_r_) was calculated use the following formula [32, 33].

$$T_{r}=\frac{4F_{max}}{\pi D^{2}}$$

Where *F*_max_ was the maximal force (N) needed to break the root and *D* was the mean root diameter. Before testing, the selected root was marked at 4 equal points, measured the diameter at each mark with a Vernier caliper, and taken the average as the root diameter.

### Hyphal length, glomalin content and water stable aggregates analysis

Hyphal length was measured according to Abbott et al. [40]. Glomalin in soil was quantified as glomalin-related soil protein (GRSP). Easily extractable glomalin-related soil protein (EE-GRSP) and total glomalin-related soil protein (T-GRSP) were measured according to the method described by Wright and Upadhyaya [25].

Water stable aggregates (WSA) were analyzed according to the method described by Wu et al. [41]. The mean weight diameter (MWD) and geometric mean diameter (GMD) were calculated using the following equations [42]:

$$\mathrm{MWD}=\sum_{i=1}^{n}{\bar{x}}_{i}w_{i}$$

$$\mathrm{GMD}=\text{exp}[\left(\sum_{i=1}^{n}w_{i}\log{\bar{x}}_{i}\right)/\left(\sum_{i=1}^{n}w_{i}\right)]$$

Where *X*_*i*_ was the mean diameter of the *i*^*th*^ sieve size, *W*_*i*_ was the proportion of the total aggregates in the *i*^*th*^ fraction, and *n* was number of sieves.

### Statistical analysis

Analysis of variance (ANOVA) and correlation analysis were performed by the program package Statistica (Version 9.1; StatSoft Inc., Tulsa, OK, USA). Fisher’s LSD was performed at *P* = 0.05 in case of significant impact by factor.

## Results

### Plant growth and mycorrhizal colonization

Twelve weeks after AM fungi inoculation, plant growth was recorded (Table 1). Both *R*. *irregularis* and *G*. *versiforme* improved plant shoot and root growth, while the effect of *R*. *irregularis* was stronger than that of *G*. *versiforme*. *G*. *versiforme* lowered plant root/shoot ratio. No mycorrhizal colonization was observed in mock-inoculated plants. The infection rate of *R*. *irregularis* was higher than *G*. *versiforme*, while both AM fungi colonized more than 70% *R*. *pseudoacacia* root after 12 weeks growth.

**Table 1:** Plant growth and mycorrhizal colonization of *R*. *pseudoacacia* 12 weeks after transplant.

Treatments	Shoot fresh weight (g)	Root fresh weight (g)	Root/shoot ratio	Colonization rate (%)
RI	24.85±1.47a	11.57±1.57a	0.46±0.05a	79.17±3.54a
GV	16.56±1.05b	6.43±0.73b	0.39±0.02b	70.83±3.76b
CK	5.74±0.35c	2.94±0.29c	0.51±0.02a	0c
inoculation	s	s	s	s

s, significant at *P* = 0.05, *n* = 6; Value with different letter indicated significant difference (Fisher’s LSD-test *P* = 0.05, n = 6); RI, inoculated with *R*. *irregularis*; GV, inoculated with *G*. *versiforme*; CK, without inoculation. Replicate data is from S1 Dataset.

### Root morphological characters and root tensile test

**Fig 1:**
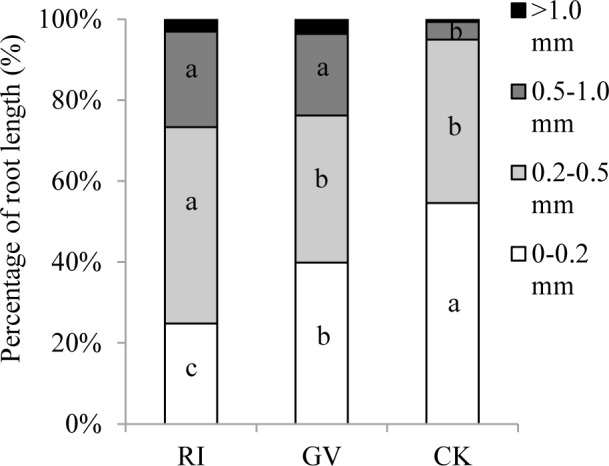
Percentage of root length in different diameter classes measured in *R*. *pseudoacacia* roots colonized by *R*. *irregularis* (RI), *G*. *versiforme* (GV) or not (CV). Significant differences are indicated by different letters (one-way ANOVA with Fisher’s LSD; *P* = 0.05; n = 6). Replicate data is from S1 Dataset.

Root length, average root diameter, root volume, number of root tip and root forks of *R*. *pseudoacacia* were improved by both AM fungi. Except the number of root tip, the improvement of *R*. *irregularis* was better than that of *G*. *versiforme* (Table 2). The percentage of root length in 0–0.2 mm diameter class in non-mycorrhizal plant (54.61%) was higher than in mycorrhizal plants (24.77% for *R*. *irregularis*, 39.83% for *G*. *versiforme*) (Fig 1). Although both AM fungi reduced the proportion in the 0–0.2 mm diameter class, root length of mycorrhizal plant in this class was still higher than non-mycorrhizal plant (data not shown). Inoculation of *R*. *irregularis* significantly improved the proportion in the 0.2–0.5 mm (48.59%), 0.5–1.0 mm (23.55%), and >1.0 mm (3.08%) diameter classes, while *G*. *versiforme* improved the proportion in 0.5–1.0 mm (20.15%), and >1.0 mm (3.61%) diameter classes, respectively.

**Table 2:** Effect of AM fungi inoculation on root morphological characters.

Treatments	Root length (cm)	Average root diameter (mm)	Root volume (cm^3^)	Number of root tips	Number of root forks
RI	1491.98±21.99a	0.53±0.01a	2.76±0.29a	909.83±56.79a	7220.67±510.87a
GV	1152.94±130.41b	0.47±0.02b	2.09±0.10b	877.17±19.71a	5394.50±486.35b
CK	644.98±20.79c	0.38±0.01c	1.01±0.09c	428.17±22.46b	3715.00±139.63c
inoculation	s	s	s	s	s

s, significant at *P* = 0.05, *n* = 6; Value with different letter indicated significant difference (Fisher’s LSD-test *P* = 0.05, n = 6); RI, inoculated with *R*. *irregularis*; GV, inoculated with *G*. *versiforme*; CK, without inoculation. Replicate data is from S1 Dataset.

For root tensile test, 80 root fragments were successfully tested for each treatment and the results were recorded (Table 3). Inoculation of *R*. *irregularis* and *G*. *versiforme* improved average tensile force and average tensile strength. Root tensile strength decreased with increasing root diameter, and their relationship can be described by a power law equation (Table 3). Inoculation of both AM fungi increased the values of parameters from the equation and hence the mycorrhizal roots were stronger in tension in this study.

**Table 3:** Effect of AM fungi on the root tensile force and strength of *R*. *pseudoacacia*, and the power law equation describe relationships between root tensile strength (*T*_r_) and root diameter (*D*).

Treatments	Average tensile force (N)	Average tensile strength (MPa)	equation	*R*^2^
RI	43.72±18.82a	21.32±2.31a	*T*_r_ = 24.288 *D*^-0.312^	0.626
GV	34.08±17.09b	18.11±2.61b	*T*_r_ = 20.517*D*^-0.357^	0.718
CK	21.10±11.97c	15.48±3.06c	*T*_r_ = 16.646*D*^-0.435^	0.735
inoculation	s	s	na	na

s, significant at *P* = 0.05, n = 80; na, not applicable; Value with different letter indicated significant difference (Fisher’s LSD-test *P* = 0.05, n = 80); RI, inoculated with *R*. *irregularis*; GV, inoculated with *G*. *versiforme*; CK, without inoculation; *R*^2^ is the goodness of fit of the model. Replicate data is from S1 Dataset.

### Hyphae length, T-GRSP and EE-GRSP in soil

**Fig 2:**
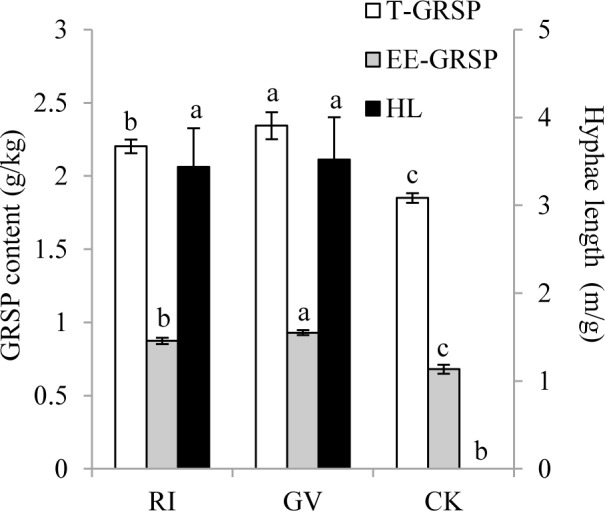
GRSP content and hyphae length in rhizosphere of *R*. *pseudoacacia* colonized by *R*. *irregularis* (RI), *G*. *versiforme* (GV) or not (CV). Significant differences are indicated by different letters (one-way ANOVA with Fisher’s LSD; *P* = 0.05; n = 6). EE-GRSP, easily extractable glomalin-related soil protein; T-GRSP, total glomalin-related soil protein; HL, hyphae length. Replicate data is from S1 Dataset.

In non-mycorrhizal treatment, no hyphae were detected (Fig 2). Inoculation of *R*. *irregularis* and *G*. *versiforme* produced more than 3 m/g hyphae in soil, but no difference was observed between two AM fungi. Inoculation of *R*. *irregularis* and *G*. *versiforme* increased the content of EE-GRSP and T-GRSP in soil, while the improvement of *G*. *versiforme* was higher than *R*. *irregularis*.

### Soil aggregate stability

**Fig 3:**
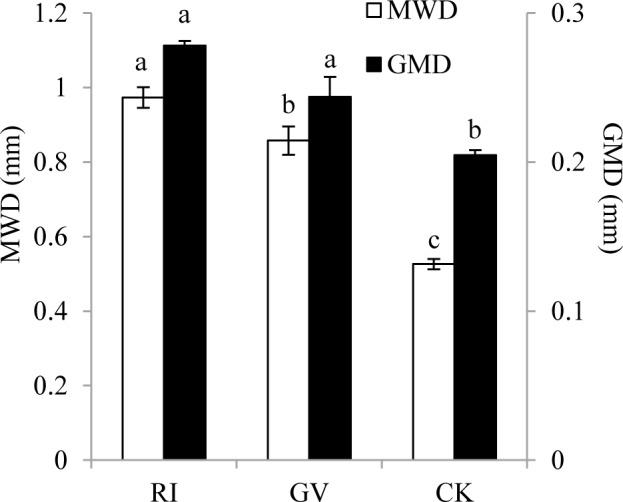
Mean weight diameter and geometric mean diameter of water stable aggregate in the rhizosphere of *R*. *pseudoacacia* colonized by *R*. *irregularis* (RI), *G*. *versiforme* (GV) or not (CV). Significant differences are indicated by different letters above the column (one-way ANOVA with Fisher’s LSD; *P* = 0.05; n = 3). MWD, mean weight diameter; GMD, geometric mean diameter. Replicate data is from S1 Dataset.

Inoculation of *R*. *irregularis* significantly increased the relative percentage of WSA_>5 mm_, WSA_5-2 mm_, WSA_1-0.5 mm_ and WSA_＞0.25mm_ fraction, while the *G*. *versiforme* only increased the relative percentage of WSA_>5 mm_, WSA _1–0.5 mm_ and WSA_＞0.25mm_ fraction (Table 4). Inoculation of both AM fungi increased the amount of WSA in soil, and the effect of *R*. *irregularis* was better than *G*. *versiforme*. Soil aggregate stability was measure through MWD and GMD (Fig 3). In this study, both MWD and GMD value were higher in AM fungi inoculated soil than in mock-inoculated soil. The improvement of MWD by *R*. *irregularis* was higher than *G*. *versiforme*.

**Table 4:** Effect of AM fungi on the percentage of soil aggregate at different size in the rhizosphere of *R*. *pseudoacacia*.

Treatment	Water stable aggregate (WSA) (%)					
	>5 (mm)	5–2 (mm)	2–1 (mm)	1–0.5 (mm)	0.5–0.25 (mm)	>0.25 (mm)
RI	6.40±0.24a	8.04±0.51a	3.72±0.44	6.03±0.15a	6.36±1.16	30.54±0.83a
GV	6.34±0.17a	5.27±1.04b	2.93±0.63	4.77±0.60b	6.65±0.06	25.97±2.09b
CK	1.67±0.22b	5.44±0.48b	3.80±0.05	3.84±0.33c	7.33±1.13	22.08±1.08c
inoculation	s	s	ns	s	ns	s

s, significant at *P* = 0.05 n = 3; ns, not significant; Value with different letter indicated significant difference (Fisher’s LSD-test *P* = 0.05, n = 3); RI, inoculated with *R*. *irregularis*; GV, inoculated with *G*. *versiforme*; CK, without inoculation. Replicate data is from S1 Dataset.

### Correlation analysis

The result of correlation analysis indicated that root length was highly correlated with WSA_＞0.25mm_, MWD and GMD (Table 5). The hyphae length in soil was highly correlated with EE-GRSP and T-GRSP content in soil. EE-GRSP and T-GRSP was highly correlated with each other, and they were not correlated WSA_>0.25 mm_ but correlated with MWD.

**Table 5:** Correlation coefficients among variables involved in GRSP content and soil aggregate.

	EE-GRSP	T-GRSP	WSA_>0.25 mm_	MWD	GMD
Root length	0.73*	0.78*	0.91***	0.96***	0.94***
Hyphae length	0.94***	0.91***	0.74*	0.93***	0.81**
EE-GRSP	1	0.92***	0.56	0.83**	0.66
T-GRSP	0.92***	1	0.63	0.84**	0.71*

EE-GRSP, easily extractable glomalin-related soil protein; T-GRSP, total glomalin-related soil protein; WSA, water stable aggregate; MWD; mean weight diameter; GMD, geometric mean diameter.

* indicate significant different at *P*<0.05.

** indicate significant different at *P*<0.01.

*** indicate significant different at *P*<0.001.

## Discussion

*R*. *pseudoacacia* is a light-demanding pioneer tree species, which was widely planted on Loess Plateau to control soil erosion [3]. In current study, both *R*. *irregularis* and *G*. *versiforme* colonized more than 70% of *R*. *pseudoacacia* seedling root at twelve weeks after inoculation. This was consistent with previous study [35]. With the well-formed symbiosis relationship, the growth of *R*. *pseudoacacia* seedling was significantly improved by both AM fungi while the improvement of *R*. *irregularis* was higher than *G*. *versiforme* (Table 1). The higher improvement of *R*. *irregularis* may be due to its fast colonization and the higher capability of phosphate transportation [43].

Root is the main organ for mineral nutrient absorption from soil. We found colonization of *R*. *irregularis* and *G*. *versiforme* changed the morphogenetic characters of *R*. *pseudoacacia* roots. Both AM fungi increased root length, average root diameter, root volume, root tips and root forks (Table 2). This was probably due to the increased root growth by the AM fungi improved nutrient acquisition [17, 18]. Besides root growth, mycorrhizal plant had a higher proportion of root in large diameter class compared with non-mycorrhizal plant, while the length of mycorrhizal plant root in the 0–0.2 mm diameter was similar with the non-mycorrhizal plant. The fine roots were usually considered in charge of nutrients acquisition [44]. For mycorrhizal plant, they could absorb mineral nutrients via not only fine roots but also extraradical mycelium [16]. For phosphate absorption, Schweiger et al. [45] indicated that root hair and AM fungi were alternative means. Although root hair was more efficient than AM fungal mycelium, the growth reliance on mycorrhizal symbiosis varied among plant species [46]. Under drought stress, although AM fungal mycelium could compensate the function of barley root hair for phosphate absorption, the root hair (unlike mycelium) improved drought tolerance was based on a different mechanism [47].

Root could increase soil shear strength by anchoring a soil layer and form a binding network in it [32]. Root tensile strength was considered one of the most important factors that influence soil stabilization and fixation, and its variation was depended on species and site factors [30]. In current study, the results of tensile test indicated that the small root had strong resistant in tension, and the strength decreased with the increasing root diameter. This was consistent with the study that compared different plant species [32, 33, 48, 49]. Effect of AM fungi on root tensile strength was first time assessed in this study, and the results indicated that both *R*. *irregularis* and *G*. *versiforme* increased the parameters value of the power law equation fit the relation between root tensile strength and root diameter. One possible explanation would be the AM fungi modified root cellulose localization and content increased root tensile strength [49, 50]. Another possibility might be the continuous inter-radical hyphae of AM fungi improved root tensile strength [16].The increased tensile strength could also due to the AM fungi induced plant genes involve in root cell wall modification [51].

Glomalin is a protein or protein class produced only by fungi from Glomeromycota, and quantified as glomalin-related soil protein (GRSP) according to the extraction methods [25–27]. In current study, both AM fungi increased the content of EE-GRSP and T-GRSP in soil at 12 weeks after inoculation. This result was consistent with the study of Bedini et al. [52], who first time confirmed the cause-effect relationship between mycorrhizal symbiosis and GRSP content. The incensement of GRSP content in current study could be explained by the contribution of extraradical hyphal length, because we found a higher correlation between GRSP and extraradical hyphal length than between GRSP and root length (Table 5). This was different with the calculation of Bedini et al. [52], in which the GRSP content had no positive relationship with total hyphal length and hyphal density. The difference might due to different host plants, AM fungi, substrate, and growth time in two studies. But Driver et al. [53] indicated that GRSP was only released into soil environment during hyphal turnover and after death of mycelia. In the study of Hallett et al. [54], mycorrhizal tomato root and AM fungal hyphae was separated in two compartments, but the increased GRSP content showed no difference. This further confirmed the study of Driver et al. [53]. Combined with the rapid turnover time of AM fungal hyphae [55], it was reasonable to speculate that the soil with higher amount of extraradical hyphae would have a faster speed in GRSP accumulation. In this way, GRSP was rather an index of former existed AM fungal extraradical mycelium than a proxy of extraradical fungal biomass [27, 52, 53].

Soil aggregate was the basic unit of soil structure and its particle size distribution and stability not only affected soil pore distribution, but also related to the storage and movement of water [56, 57]. In previous study, inoculation of AM fungi could increase the fraction of WSA in soil [58]. Similarly, inoculation of *R*. *irregularis* and *G*. *versiforme* in current study increased the amount of WSA_>0.25 mm_, MWD and GMD. In soil growing mycorrhizal plant, AM fungi could hold soil particles together via physical entanglement by its extraradical mycelium formed skeletal structure, and this favored the formation of micro-aggregates and smaller micro-aggregates into macro-aggregate structures [28]. Besides extraradical mycelium, AM fungi produced GRSP could also bind micro-aggregates into macro-aggregates through a ‘gluing’ action and change the distribution pattern in WSA sizes [26, 59–60]. In current study, we found hyphae length had a stronger effect on soil aggregate than the content of GRSP (Table 5). This result was inconsistent with the study of Rillig et al. [61] who indicated that direct effect of GRSP was stronger than direct effect of AM fungal hyphae in a field test, but consistent with the result of Bedini et al. [52] who suggested AM fungal mycelial network may directly affect soil aggregates stability in a pot test. Although the inconsistency may due to the value used for soil aggregates stability analysis the influence of plant species on soil aggregate evaluation could not be neglected. Combined with the effect of AM fungal hyphae and GRSP, plant root length also contributed to the formation of soil aggregates because we found root length was highly correlated with WSA_>0.25 mm_, MWD and GMD (Table 5). The influence of plant root on soil aggregates formation could be explained by (1) root physical force/penetration; (2) soil water regime alteration; (3) rhizodeposition; (4) root decomposition; and (5) root entanglement of soil particles, and AM fungi involved in all these process [61–63].

With the help of AM fungi, the performance of *R*. *pseudoacacia* in soil stabilization and fixation as well as drought tolerance should be promoted [28, 30, 57]. And this opened the possibility to use AM fungi in *R*. *pseudoacacia* plantation on Loess Plateau. However, due to the different environment conditions, field experiments are still needed to verify the effect of AM fungi on the performance of *R*. *pseudoacacia*.

## Conclusions

Our work confirmed that *R*. *irregularis* and *G*. *versiforme* could form symbiosis with *R*. *pseudoacacia* seedling in pot condition. With the establishment of symbiosis, both AM fungi could (1) promote the growth of *R*. *pseudoacacia* and change the morphological characters of roots, including the proportion of root in different diameter, root length, average root diameter, root volume, root tips and root forks; (2) strengthen root tensile strength; (3) increase the content of EE-GRSP and T-GRSP in soil; (4) improve soil aggregate stability.

## Supporting Information

S1 Dataset Data used in this study (xlsx file). (XLSX)
